# Overestimation of infarct size following acute myocardial infarction is related to extent of myocardial edema

**DOI:** 10.1186/1532-429X-16-S1-O21

**Published:** 2014-01-16

**Authors:** Ananth Kidambi, Akhlaque Uddin, David P Ripley, Adam K McDiarmid, Peter P Swoboda, Tarique A Musa, Bara Erhayiem, Gavin Bainbridge, John P Greenwood, Sven Plein

**Affiliations:** 1 Cardiology, Multidisciplinary Cardiovascular Research Centre & The Division of Cardiovascular and Diabetes Research, Leeds Institute of Genetics, Health & Therapeutics, University of Leeds, Leeds, UK

## Background

Late gadolinium enhancement (LGE) is an accurate and reproducible method to delineate nonviable myocardium following myocardial infarction (MI). However, in the early stages following acute MI, LGE has been shown to overestimate the size of the infarct zone by up to 30%. The causes for this are unclear, and may be related to tissue remodelling, intracellular contrast uptake, or expansion of the interstitial space. Myocardial edema is a feature of reperfused acute MI, and edematous myocardium has been associated with early contrast enhancement [1]. We hypothesised that the presence of tissue edema is also related to late enhancement, and contributes to overestimation of infarct size in acute MI.

## Methods

46 patients received CMR examination at 3.0T at 2 days following reperfused ST-elevation acute MI, with follow-up imaging at 10 days and 3 months. Short-axis T2-weighted imaging and cine imaging were performed, as well as LGE imaging 16-20 minutes following administration of 0.1 mmol/kg gadolinium DTPA. Edema volume was measured on T2-weighted imaging and scar volume measured on LGE imaging, both quantified using a semi-automated histogram-based thresholding method (Otsu method). The change in scar volume was compared to the change in edema volume between day 2 and day 10, and between day 2 and 3 months.

## Results

**Figure 1:**
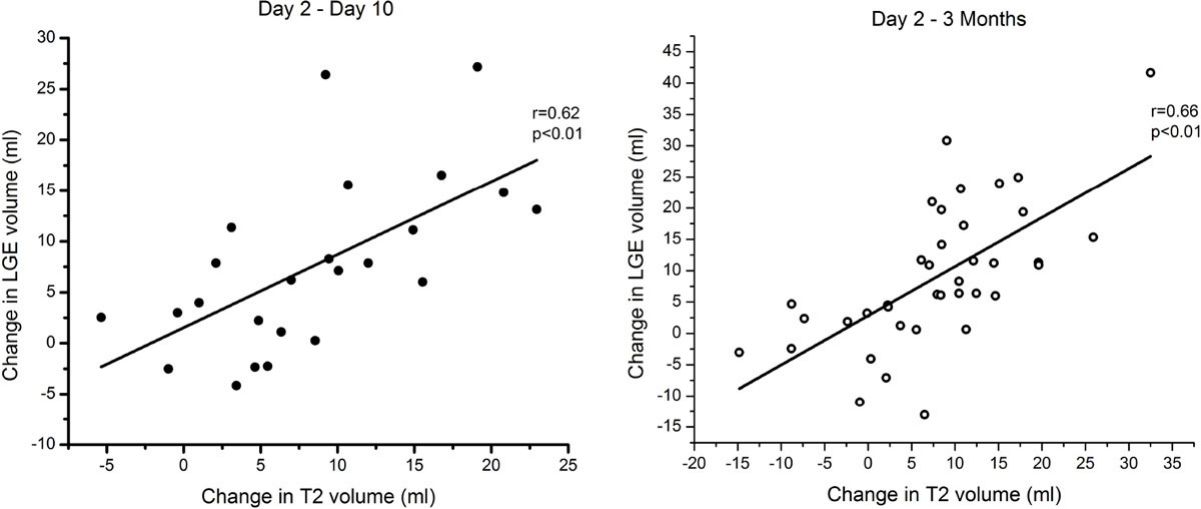
**Change in estimation of scar volume by LGE correlates with resolution of myocardial edema by day 10 (left panel) and 3 months (right panel)**.

**Figure 2:**
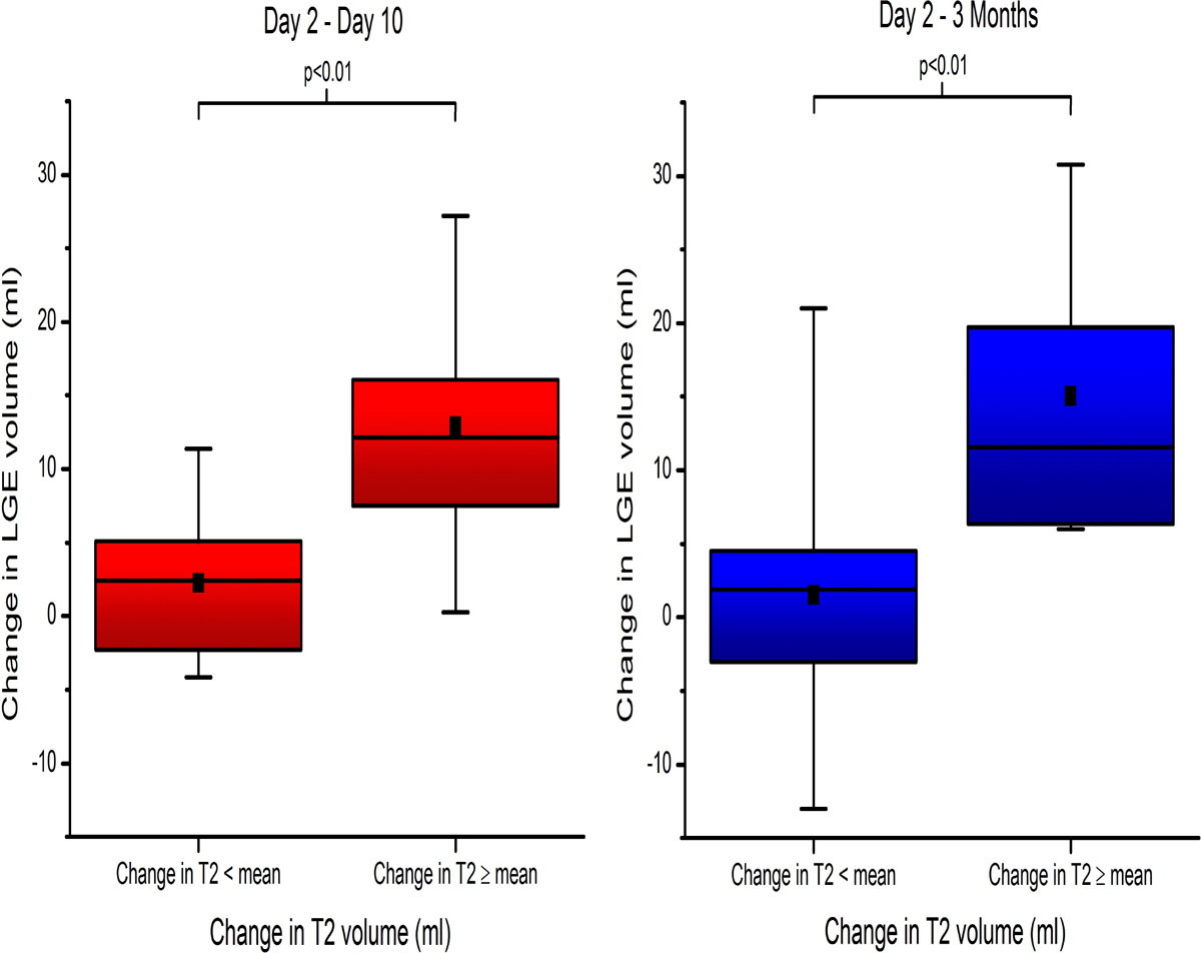
**Acute overestimation of scar volume is related to resolution of myocardial edema by day 10 (left panel) and 3 months (right panel)**.

Of the 46 patients studied, 27 (59%) completed imaging at day 10 and 39 (85%) at 3 months. Mean scar volume decreased by 7 ml (23%, p < 0.01) at 10 days and 9 ml (28%, p < 0.01) at 3 months. There was significant correlation between change in edema volume and change in scar volume from day 2 to day 10 (r = 0.62, p < 0.01) and day 2 to 3 months (r = 0.66, p < 0.01) (Figure 1). Stratifying patients into two groups based on mean change in edema volume, patients with more change in edema had significantly higher change in scar volume (Figure 2).

## Conclusions

LGE CMR overestimates scar volume acutely following acute reperfused MI. This overestimation correlates with the volume of myocardial edema detected acutely.

## Funding

JPG and SP receive a research grant from Philips Healthcare. SP is funded by British Heart Foundation fellowship (FS/10/62/28409).
